# Social Modeling and Eating Behavior—A Narrative Review

**DOI:** 10.3390/nu13041209

**Published:** 2021-04-07

**Authors:** Julia Suwalska, Paweł Bogdański

**Affiliations:** Department of Treatment of Obesity, Metabolic Disorders and Clinical Dietetics, Poznan University of Medical Sciences, Szamarzewskiego 82/84, 60-569 Poznań, Poland; pbogdanski@ump.edu.pl

**Keywords:** social influence, social matching, mimicry, food intake

## Abstract

Social modeling of eating is the adjustment of the amount of food eaten to the intake of the accompanying person. In this paper we provide a narrative review of literature on social modeling of eating with a particular focus on recent studies. Firstly, we describe the structure of a typical modeling experiment. Secondly, we present a variety of research in this field: experiments with various types of confederates, experiments aimed at the evaluation of the influence of gender, partner’s body weight, type of food, hunger, personal characteristics, etc. Thirdly, we present practical implications of this knowledge. The common conclusion is that social modeling of eating occurs in different situations and consumption is adapted to the standards established by the eating partner, but is not their direct reflection. Social influence of eating is not restricted to "artificial" laboratory situations; social modeling and social norms manipulations may be used to change people’s dietary practices, especially in children and young adults. Within the home environment parental modeling has been shown to promote children’s snacking and fruit and vegetable consumption. Social modeling may be used in nutrition interventions aimed at the improvement of children’s diet and in obesity prevention programs.

## 1. Introduction

Eating, like smoking and drinking alcohol, is an activity strongly influenced by social context [1]. People eat differently depending on whether they are eating alone or with someone, whether a stranger or a loved one, a business meeting or an evening out with friends. Although eating is largely regulated by hunger and satiety, the presence of other people also has a significant impact as it serves as a social norm. Social norms in general are indications of what is appropriate to do in a particular situation. In the case of situations involving eating, they are indications of how much is appropriate to eat [2]. Herman, Roth, and Polivy [3] described three primary mechanisms driving the influence of the presence of others on food intake: modeling, social facilitation, and self-presentation. Social modeling occurs when individuals eating in the presence of models who consistently eat a lot or a little, likewise tend to eat a lot or a little. To put it differently, people eat less when others eat a small amount and vice versa [4]. The social facilitation theory describes situations where people in a group, especially acquaintances, eat more. The theory of self-presentation states that if people eat in the presence of people who they believe are watching them and judging their intake, they eat less than they would have eaten if they were alone [3].

The aim of this study is to present a variety of experiments in the field of social modeling of eating with a particular focus on recent studies and practical implications of this knowledge.

## 2. Overview

In this paper we provide a narrative review of literature on social modeling of eating. Firstly, we describe the structure of a typical modeling experiment. Secondly, we present a variety of research in this field: experiments with various types of confederates, experiments aimed at the evaluation of the influence of gender, partner’s body weight, type of food, hunger, personal characteristics, etc. Thirdly, we present practical implications of this knowledge.

A few reviews of social modeling of eating have been published. The previously mentioned review by Herman et al. [3] published in 2003, focuses on different effects of the presence of others on food intake. A meta-analytic review by Vartanian et al. [5] presents articles published till the end of 2013 while Cruwys et al. [6] have reviewed articles on social modeling of both food intake and food choice published between 1974 and 2014.

In our narrative review, we present descriptions of a variety of social modeling experiments. Such detailed descriptions are needed to fully explain this robust phenomenon to practitioners new to this topic. Review can help both experts and non-experts to make sense of the increasing volume of original publications [7] and help researchers and policymakers to identify gaps in the knowledge [8].

We also provide an up-to-date review of experiments published between January 2015 and February 2021 which have not been covered in the abovementioned reviews. To assess this, Scopus, PubMed, and Web of Science databases were searched using the following keywords: social modeling or matching and eating or food intake. We also screened references of the selected articles. Only empirical studies measuring participants’ intake in the response to social cues (partner’s intake) published in English were included. Fifteen articles published in 2015 and later matched the criteria and are included in the review. The general characteristics of the selected studies are presented in Table 1.

## 3. Structure of a Typical Modeling Experiment

In a nutshell, the real purpose of research into social modeling is to assess participant’s food intake in response to another person’s food intake. Those experiments often take place in laboratory settings—e.g., at universities. It should be remembered that when participants know that they are being watched and the amount of food is measured, their consumption is affected. The respondent is supposed to behave as naturally as possible, rather than focusing on the element of their behavior that is being studied [43]. Therefore, in research, deception is used in the form of a cover story and cover task which conceal the real aim of the experiment. A cover story is false information about the entire study, a cover task is the specific task to be performed by the study participant. An example of a cover story is informing the participants that they take part in a food tasting test [10] or evaluate advertisements [22]. An example of a cover task can be solving a jigsaw puzzle [39]. At the same time, food is available for participants to consume and subsequently, the amount eaten is evaluated. In some studies, after the experiment has been completed, participants are asked to indicate what they think the purpose of the study was. The analysis does not take into account the results of participants who indicated the actual purpose of the study (assessing factors that influence consumption).

A common feature of a majority of modeling studies is the participation of an experimenter’s helper, referred to as a confederate. This is a person claiming to be a participant, but actually employed in the study and following researchers’ instructions [2]. The experimenter’s helper usually influences the behavior of the participants in the experiment. The confederate may deliberately mislead them, cause them to take certain actions. Investigation of participants’ reactions to the situation “provoked” by the confederate is the real goal of the experiment. In some studies, instead of participation of a confederate, the experimenter provides information to influence the participants, e.g., specific data presented as the consumption rate of previous participants (remote confederate). However, in some studies there is no confederate. Instead, a pair of participants, who did not receive instructions on how much to eat take part in an experiments and form “a dyad”. The amount of food consumed by each of participant in a dyad is subsequently measured [38].

A pioneering study on the effect of the presence of others on eating was conducted in the 1970s by Nisbett and Storms [9]. Participants were informed that they were taking part in a cracker tasting test. Some of them ate with a confederate, tasked with eating a certain number of crackers, either small or large. The other participants ate alone (control group). The aim of the study was to assess the number of crackers eaten and its determinants. Researchers found that the number of crackers eaten by the confederate had a significant effect on participants’ behavior. If the confederate ate a lot, participants ate more than those in the control group, if little, they ate less than the control group.

Since then, many experiments have been conducted on the effect of the amount of food eaten by the confederate on the participants’ intake. Selected studies best presenting variety of situations and conditions in which social modeling occurs or not will be described below.

## 4. Influence of Knowledge of Previous Participants’ Intake

In a study by Roth et al. [10], information about the consumption of a remote confederate was used for the first time. In their experiment, 134 female participants agreed to take part in a cookie taste test. They were told that their task was to evaluate the taste of three types of cookies and that they could eat as many of them as they wanted. The participants were randomly assigned to one of three groups—low or high consumption or a control group. In two experimental groups, a piece of paper was left on the table with false information about the number of cookies eaten by previous tasting participants. The participants were also informed that this was technical information for the experimenter. In the low consumption group the numbers were low (3–5 per person), in the high consumption group they were high (13–15 per person). In the control group there was no such information. Consistent with the study design, participants in the high intake group ate significantly more cookies than those in the low intake group and the control group. The information about the behavior of previous participants given to the test subjects therefore represented the influence of a remote confederate.

This study design was also used by Vartanian et al. [11] and Robinson, Benwell, and Higgs [12], among others, who obtained similar results. In the study by Feeney et al. [13], participants were randomly paired with either a confederate or a remote confederate. The effect of social modeling was observed to be of similar intensity in both groups. 

## 5. Influence of a Recording Involving a Confederate

Studies using video-confederate recordings have not provided conclusive results. Romero, Epstein, and Salvy [14] assessed whether girls would adjust the number of cookies eaten to match the intake of a peer in the recording who ate a low or high number of cookies. The cover task was a card sorting test (performed by both the participants and the girl in the video). Participants who watched a video with a large portion of cookies ate significantly more cookies than those watching a video with a small portion. 

Different results were obtained by Hermans et al. [15] in a group of young women. In the first experiment, the participants watched one of two films whose protagonist was a woman doing office work. In the first version she ate a few jelly beans while working, in the second she ate nothing. In the second experiment, the subjects watched one of three films in which the woman was talking on the phone while sitting on the couch and, depending on the version, ate nothing or had a small or large portion of M&M’s. In both experiments, subjects were allowed to eat M&M’s chocolates while watching. In neither experiment was there any modeling of the participants’ consumption to that of the woman in the film; the amount of candy consumed by the participants was similar and independent of that observed in the film. 

In a more recent study of McGeown et al. [16] under the guise of rating empathy, 93 female participants viewed a female video confederate "incidentally" consume either a low or high number of chips while watching a movie. They were also informed that they should feel free to help themselves with chips which were left on the table in front of them. The results showed that participants’ consumption of chips was positively correlated with the model’s consumption.

In the study of Romero, Epstein, and Salvy [14], the peer in the video selected the same cookies in the same room as the participants in the study. Similarly, in the experiment of McGeown et al. [16], participants watched a protagonist watching a movie and had the same snack as hers. In contrast, in the Hermans et al. [15] study, the room and the task performed were different from those of the participants. Thus, this may have affected the perception of the female protagonist in the video as an indicator of consumption and caused the modeling to not occur. 

## 6. Influence of Gender

The majority of social modeling studies have been conducted with female participants. This is partly due to the fact that experiment participants are often students of psychology, the majority of whom are female [2]. The results of studies assessing the effect of participants’ gender on modeling are inconclusive and should be treated with caution [6]. However, there is some indication that the impact of social modeling in men may be weaker than in women [17]. The difference between men and women may be due to the fact that for women the way they are perceived is more important than for men and they feel obliged to fit in with the other person [44]. Further research into gender differences in social modeling is needed. 

## 7. Influence of Body Weight of the Confederate 

Several studies have assessed whether the body weight of the confederate matters in the social modeling process. In a study by Johnston [18], subjects participated in an ice cream taste test in the presence of a confederate who (as instructed by the researcher), ate a small or a large amount of ice cream. An additional variable was the body weight of the confederate—normal versus obese. In the case of a normal-weight confederate, the modeling effect was evident—subjects ate a lot if the confederate ate a lot, and ate little if the confederate ate little. However, when the confederate was obese such a relationship did not occur—participants ate little ice cream regardless of the amount consumed by the confederate. 

In a study by McFerran et al. [19], participants were given the option of serving candy to themselves before screening a short film and eating it while watching. They were paired with a confederate who took either 2 candies (indicating a low intake standard) or 30 candies (indicating a high intake standard). Two options were used: a slim confederate and an obese confederate (the same person in a specially designed costume). The overall conclusion of the study was that the subjects ate little or a lot adapting to the confederate, indicating a significant role for social norms. Significant in the authors’ opinion was the effect of the confederate’s body weight on the subjects’ behavior. When confederates set a high standard—the participants ate less when they were an obese person. Conversely, when they set a low standard (ate little candy) and were obese, participants consumed more. Thus, a difference in behavior does not always mean a reduction in intake.

Hermans et al. [21] assessed the difference in consumption of M&M’s candy by young women of normal body weight. They were paired with a slim or normal weight confederate (the same confederate, normal weight was obtained by using a costume) who ate nothing, ate little or ate a lot. Social modeling only occurred when the confederate was a normal weight person. The researchers suggest that young women of normal body weight are more likely to mimic the amount of food consumed by the confederate when the subjects’ and confederate’s body weights are similar. 

Robinson et al. [20] tested the effect of exposing female participants to information about the food intake of either normal weight or overweight individuals. Eighty females were invited for a cookie taste test and were provided with an either small or large intake information about normal weight or overweight previous participants (remote confederates). Regardless of the weight-status of the remote confederates, participants ate more food when they believed that previous participants had eaten a large amount of food, in comparison with when they believed previous participants had eaten a smaller amount of food. In opposition to results of previously mentioned studies, researchers concluded that women may model the food intake of other women, even when they believe they are of a different weight status to themselves.

The studies described here show that the body mass of the confederate can influence the amount of food consumed by the participants. It is indicated that the effect of social modeling on food intake can be inhibited when the confederate’s body mass shows deviations from the norm [33]. However, having only information about body mass of other people, without actually eating with them (remote confederates), might not be sufficient to influence food intake of the participant.

## 8. Influence of Type of Food 

Energy-dense snacks (chocolate, and biscuits) are typically used in social modeling studies. Hermans et al. [22] have investigated whether social modeling also occurs when healthy snacks are available. Participants were told that their task was to evaluate advertisements (cover task), and they were given an opportunity to treat themselves to vegetables during breaks. Each of them was paired with a confederate who ate nothing, ate little or ate a lot. It was found that when the confederate ate a lot of vegetables, the participant ate more than when the confederate ate little or nothing, indicating that the modeling process also occurred when vegetables were consumed. 

In a study of Stel et al. [23] researchers assessed healthy food consumption depending on confederate’s body weight appearance. In Study 1, participants watched a short film fragment together with a confederate who appeared normal weight or overweight and consumed either 3 or 10 cucumber slices. In Study 2, a confederate who appeared underweight, normal weight, or overweight consumed no or four cucumber slices. Results showed that participants’ healthy eating behavior was influenced by the confederate’s intake regardless of confederate’s body weight.

Similar results were obtained by Liu and Higgs [24], in whose study participants took part in a vegetable taste test (cucumbers and red peppers) with or without information about the large intake of previous participants (a remote confederate). Subjects in the group with information about the remote confederate ate significantly more than those who did not receive this information. 

Pliner and Mann [25] assessed the effect of food palatability on social modeling. Participants were divided into two groups: one had palatable food and the other had less palatable food. In each group, the experimental subgroup had been provided with information whether previous participants had eaten a lot or a little (a remote confederate) and the control subgroup had no such information. In the group with tasty food, the effect of social modeling was observed—subjects ate more when they had information about high consumption of the remote confederate. In the group with less tasty food, social modeling did not occur.

In a recent study of Kimura et al. [26] researchers assessed whether partner’s presence affects unfamiliar food intake. In the experiment, undergraduate students were invited to take part in “sensory evaluation of various snacks” and could participate either alone or with a friend. Participants were asked to taste pieces of 13 kinds of snacks, including three unfamiliar and ten neutral snacks, and were informed that they did not have to eat a snack if they did not want to. The total amounts and ratios of all kinds of snacks consumed for unfamiliar and neutral snacks were compared between participants in the pair condition and those in the individual condition. Results demonstrated that the ratio of participants who consumed all three kinds of unfamiliar snacks was higher in the pair condition than in the individual condition.

Social models exert a powerful influence on eating behaviors. They may be employed to improve the ability to adequately regulate food intake and to promote the consumption of healthy and unfamiliar foods [5,26]. However, the results of Pliner and Mann have shown that although it is relatively easy to induce people to increase consumption of palatable foods by means of social influence, it is not so easy to get them to increase their consumption of unpalatable foods by the same means [25]. Therefore, interventions aimed at increasing healthy food intake should take account of individual’s taste preferences. 

## 9. Influence of Hunger

Goldman, Herman, and Polivy [27] assessed whether a strong feeling of hunger would make participants resistant to the social influence of a partner. In the first experiment, participants were divided into three groups. Those from the first group were asked to abstain from food for 4 h, and immediately before the test they were given a milkshake (to partially satisfy their hunger). In the second group the fasting period was 12 h and in the third group it was 24 h. The subjects were given a cover story that they would be taking part in a hunger and taste sensation test and that the test consisted of two parts, one at lunch time and one in the afternoon, with no food allowed in between. During lunch, the participants evaluated the taste of the food (sandwiches, fruit, and biscuits) and then were allowed to eat without restriction. A confederate also participated in the study and was tasked with eating an amount of food set by the experimenter. Interestingly, the participants, regardless of the severity of their hunger, adapted the amount of food to that eaten by the confederate—when she ate little, they too ate little, when she ate a lot, they also ate a lot. 

Similar results were obtained in the second experiment. The behavior of two groups was compared: in the first group the participants did not eat for 24 h, in the second group there was no food ban before the experiment. The study was conducted in two configurations: the participants were paired with a confederate or ate alone. Here, too, there was an adjustment of food intake to that of the confederate, regardless of the degree of hunger. According to the researchers, social modeling has a stronger influence on eating than the feeling of hunger and satiety. 

A study by Hermans et al. [17] assessed social modeling in a group of 59 young men paired with a confederate (also male) who ate nothing, ate little, or ate a lot of peanuts in between watching and rating videos (cover story). After the test, participants were asked to rate their hunger before the experiment on a scale of 1 to 10 and to state how many peanuts the confederate had eaten. Only men who were hungry at the beginning of the experiment adjusted the amount of peanuts they ate to the consumption of the confederate—they ate more when he ate more, they ate less when he ate less. Hunger may therefore play an important role in the social modeling of young males. The researchers point to two possible reasons for this relationship. The first is that men need to be hungry for social modeling to occur. Their hunger made them want to eat as much as possible to meet their body’s needs, but they still paid attention to the confederate’s intake so that they did not eat much more than he did. Men who rated their hunger level as low or medium had no incentive to eat as much as possible, so even when the confederate ate a lot, they did not eat more (and modeling did not occur). The first explanation therefore suggests that hungry men eating with a confederate who ate little reduced the amount of food they would have eaten. The second explanation is that men who ate with a high-consumption confederate ate more because their hunger was compounded by additional visual and auditory stimuli—the sight of peanuts, the crunching sounds made by the confederate. Hungry subjects were much more accurate in indicating how much the confederate had eaten, which indicates that they were more sensitive to intake rates. 

## 10. Influence of Personal Characteristics

Research of Hirata et al. [28] focused on the influence of informational eating norms on people’s food intake, and examined whether this influence was moderated by participants’ self-construal levels. This concept refers to one’s self-views, or the extent to which a person defines herself independently of others or interdependently with them. The independent self-construal is characterized by a focus on autonomy and distinctiveness from others, whereas the interdependent self-construal emphasizes social bonds, group memberships and social relationships for self-definition [45]. Researchers conducted two experiments, one in Brazil and one in Germany. Firstly, participants were left alone to activate the independent or interdependent self-construal. To do so, participants were asked, with an open-ended question, either to report what they had in common with (for interdependence) or what differentiated them from (for independence) their close friends and family. Directly after this priming procedure, participants were asked to indicate how much overlap they felt with their close friends and family. Subsequently, participants were exposed to either the low- or high intake norm of remote confederate and a bowl of chocolates. In experiments in both countries social modeling occurred, but self-construals did not moderate modeling effects on food intake. Researchers concluded that Brazilian and German female young adults are vulnerable to modeling effects on food intake, independent on their self-construal. In another study Hirata et al. [29] found that modeling occurs regardless of participants’ self-esteem.

In previously mentioned study by Liu and Higgs [24] researchers investigated whether modeling of food intake is moderated by strength of identification with the norm referent group. In two conducted studies remote confederate design was used. Additionally, students completed a questionnaire on their identification as a Birmingham student. Modeling effects were found across both studies but the extent to which the participants identified as a Birmingham University Student did not moderate these effects. 

Reid et al. [30] tested whether self-affirmation reduces conformity to unhealthy behavior (eating unhealthy food). To assess self-affirmation, participants ranked 11 characteristics and values (e.g., relationships with friends and family, or humor) from most to least important. Self-affirmed individuals wrote about why their first-ranked value was important to them. Individuals who were not self-affirmed wrote about why their ninth-ranked value might be important to someone else. In the taste perception section of the study, the researcher presented the peer modeling sheet (remote confederate), the bowl of cookies, and the taste rating form to participants. The results of the study showed that participants modeled food intake and self-affirmation did not reduce this tendency. 

Bischoff et al. [31] conducted a study using a video-confederate to assess the impact of temporal distance on snacking. The cover story was a pretzel taste test. Firstly, the participants were asked to imagine their lives in one day (proximal) or in one year (distant condition). Afterwards, they watched a video of a protagonist choosing one of two brands of pretzels and eating either plenty or a few of them. Then, participants ate as many pretzels as they liked while filling in a tasting questionnaire. Participants primed with proximity imitated snack intake more than participants primed with distance. Researchers suggest that self-distancing from the situation may help to stick to a diet and reduce the risk of overeating while eating with a person who eats a lot. 

In 2020, a meta-analysis of research Vartanian et al. [32] tested whether there is a difference in social modeling between restrained (chronic dieters) and unrestrained eaters. The group consisted of 735 female participants—data combined from 8 experimental studies (5 with remote confederate and 3 with live confederate). All of the studies included had three norm conditions: a high-intake norm, a low-intake norm, and a no-norm control and used the Restraint Scale to measure dietary restraint. Restrained and unrestrained eaters did not differ in how much they ate in the low-intake norm condition, and there was also no significant difference between restrained and unrestrained eaters in the magnitude of the inhibition effect. Across studies, restrained eaters ate more than unrestrained eaters did in the high-intake norm condition, and also showed a substantially larger augmentation effect than did unrestrained eaters. Researchers concluded that social norms provide an upper limit for acceptable food intake, with high-intake norms permitting (but not requiring) individuals to indulge themselves. In researchers’ opinion, the fact that restrained eaters were more responsive to the high-intake norm than unrestrained eaters suggests that the high-intake norm gives restrained eaters permission to indulge when they typically eat less than they want to.

## 11. Persistence and Resistance to Social Norms of Eating

Feeney et al. [36] conducted a study on the resistance to, and persistence of, a social modeling. In the experiment participants for four consecutive days watched the television show and could help themselves with pizza. In the persistence section participants were paired with remote confederates during first session and for the remaining three sessions, ate alone. In the resistance section ate alone until their final session, at which time either a high or low norm was presented. The results showed that when participants ate alone following a session with norm-setting remote confederates, the effect of the social influence persisted. However, the persistence effect varied by norm and weakened over time. Participants modeled a low eating norm for only one additional session and the size of the effect was markedly weaker. By contrast, the high norm persisted for all of the remaining sessions. Thus, individuals’ social influence histories can affect their eating.

To gain more insights into the persistence of effects of social modeling, Polman et al. [37] explored how food intake of young women changes as a result of previous exposure to a non-eating confederate. In a study, participants were given access to chocolates at two different time points. First, participants were all paired with an unfamiliar non-eating confederate. Afterwards, half of the participants remained with the non-eating confederate, while the other half was left alone with the food. Results indicated that participants who were left alone increased their intake on average. In contrast, most of the participants who remained with the non-eating stranger did not increase intake. Researchers concluded that if intake behaviors are too extreme and divergent from the desire to eat as much as possible, women may, on average, only adhere to these behaviors in the presence of others.

## 12. Influence of Other Factors

Brunner [33] conducted two experiments in which subjects participated in chocolate taste test in the presence of a confederate who ate a small or large amount. The effect of external cues on modeling was assessed. In one group, a TANITA scale stood in the room where the tasting took place. In the second experiment, when placing a bowl of chocolate in front of the participant the experimenter said: "chocolate makes you fat but happy". Some participants were a control group, with no external cues. Social modeling only took place in the control group. In both experimental groups, participants ate little regardless of the consumption standard set by the confederate. The results of the study therefore indicate that external cues are important for eating behavior. 

In a study by Bevelander et al. [34], primary school children watched one of three movies (happy, neutral, or sad) with a same-sex, normal-weight confederate who was asked to eat nothing or to eat a specified number of snacks (10 chocolate-covered peanuts). The children who watched the sad or happy film were more likely to adjust their snack intake to match the confederate. The researchers suggest that children eat more inattentively when they watch an emotional film, and therefore respond more automatically to a peer’s snack intake. However, when they watch a neutral film, they are less sensitive to peer’s intake. The researchers stress that children have no control over the environment in which they eat, so parents should keep children’s eating in front of the TV to a minimum. 

Kaisari et al. [35] investigated whether familiarity of dining partners affects modeling. In two studies, female dyads completed a task together whilst having access to high energy dense snack foods. Modeling was observed regardless of the familiarity of the dining partners and food types consumed.

## 13. Behavioral Mimicry

Hermans et al. [38] conducted a study on the importance of behavioral mimicry. Study participants in pairs consumed a meal and researchers analyzed each bite of food. Bites were considered behavioral mimicry if they occurred within 5 s of their partner’s bite. It was found that the participants mimicked each other’s way of eating by adjusting their pace to that of their partner. They were therefore more likely to take bites in unison than each at their own pace.

Similar results were obtained in a study by Bevelander et al. [39], in which the study group consisted of children paired with a confederate—a same-sex peer of normal weight. The cover task was a jigsaw puzzle. The children were allowed to treat themselves to chocolate-coated peanuts, and the confederate’s task was to eat one peanut every minute (10 peanuts in a 10-min experiment). As in the study by Hermans et al. [38] described above, it was analyzed whether children imitated the confederate’s eating and ate the nuts within 5 s after or independently of the confederate. Results indicated that imitation occurred and the sight of a peer reaching for a snack encouraged the child to do so.

In a research of Sharps et al. [40] videos of thirty-eight parent and female adolescent dyads eating a lunchtime meal together were examined. The study assessed whether a parent placing a food item into their mouth was associated with an increased likelihood that their adolescent child would do the same. Parents’ and adolescents’ overall food intake was positively correlated, whereby a parent eating a larger amount of food was associated with the adolescent eating a larger meal. Results showed that adolescent females mimicked their parental eating behavior, selecting and eating more of a food item if their parent has just started to eat that food.

According to the authors of abovementioned publications, mimicry may be one of the causes of social modeling. The sight of a person reaching for food encourages both adults and children to eat. This may form the basis for interventions to reduce overeating or increase the intake of valuable foods, e.g., by preparing healthy snacks for children [39]. 

## 14. Awareness of Social Impact

In the previously mentioned study by Roth et al. [10], participants tasted cookies given information about how many had been eaten by previous participants (participation of a remote confederate). At the end, they were asked the reason for consuming a given number of cookies. Most often they mentioned hunger or satiety, palatability, and tasting requirements. No participant mentioned the influence of information about other participants. Only 12% of the participants indicated reasons related to how they would be perceived. 

In 2013, Vartanian et al. published the results of three experiments [11]. Two of them were similar to the study of Roth et al. [10] and involved the participation of a remote confederate. In the third experiment, each participant was solving a task while having access to sweets. At the same time, the task was being solved by a confederate who ate a lot or a little of the sweets. In each experiment, subjects were asked to judge what influenced the amount of candy eaten (i.e., hunger, taste, and amount eaten by a confederate or remote confederate) and how much was appropriate to eat. Participants paired with the confederate or remote confederate who ate little indicated a smaller amount as appropriate to eat. They also ate statistically less than those paired with a confederate with a high intake. Although social modeling was observed in all experiments, subjects were significantly more likely to give taste and hunger than social influence as reasons for eating sweets. 

These findings were consistent with a previous study by Vartanian et al. [41], in which participants watched television in pairs while given slices of pizza, and then rated what influenced the amount of food they consumed. The most common answers were hunger and satiety, taste, time since last meal, and watching television. Although there was a clear influence of the amount of food consumed by the partner, only 2.5% of participants indicated social influence.

Spanos et al. [46] conducted two experiments testing awareness of social influence. In the first experiment, participants watched one of six short videos in which one or two people performed tasks on a computer while having access to popcorn. In different versions of the video, the protagonist ate alone, the two people ate independently, ate the same amount, or one person mimicked the other’s consumption (eating a popcorn within 5 s). Subsequently, the subjects rated what influenced the popcorn consumption of the movie characters. Results indicated that participants correctly identified social influences on eating, especially when one person mimicked the other’s consumption pattern. 

The second experiment by these authors was different in nature. According to the cover story, the participants’ task was to watch a film with another person and discuss their experiences. M&M’s chocolates were at the viewers’ disposal (both during the screening and the discussion). The task was performed in pairs—each participant was paired with a confederate who consumed little or a lot of candy. This part of the experiment was recorded. In the second part, the recording was replayed to participants to refresh their recollection of the interaction with their partner. The sound track of the recording was turned off so that participants focused on their own and the confederate’s behavior rather than on the content of the video or the discussion. After watching the recording, the subjects analyzed what influenced the amount of candy they consumed during the experiment. Participants also completed a Social Eating Scale questionnaire about the influence of others on their eating behavior in everyday life—including whether they eat a lot when others eat a lot, and whether they feel like eating when others eat. Only those who scored high on the Social Eating Scale correctly identified the influence of a confederate on how much candy they consumed. The others did not perceive a social influence on their consumption. 

The results of the study of Robinson et al. [42] showed that of the 160 participants, 34% reported that they had been influenced, 10% were unsure and 56% reported they had not been influenced. Crucially, participants’ reports of social influence appeared to be accurate; the food intake of participants reporting social influence was significantly affected by the amount of food other people had been eating, whereas the food intake of participants denying social influence was unaffected. Researchers concluded that individuals may be more aware of the effect that social influence has on their eating behavior than previously assumed.

As the studies mentioned above indicate, people are often unaware of the influence of other people on the amount of food they consume. Increasing awareness of social influence on food intake, especially high-calorie snacks, could be used to promote a healthy lifestyle and weight loss process. In addition, one’s eating partners can perhaps be chosen more judiciously. In this way, people can use external environmental cues to “unconsciously” eat better [41].

## 15. Discussion

The studies presented in this review do not cover the entirety of experiments in social modeling of eating; rather, they indicate directions of research in this field and their, sometimes contradictory, results. Undoubtedly, the common conclusion is that social modeling of eating occurs in different situations. Consumption is adapted to the standards established by the eating partner, but it is not a direct reflection of these standards. The extent to which people adjust their intake is influenced by a number of factors, including: the gender and age of the subjects, the type of snacks, internal (e.g., hunger, or personal characteristics) as well as external factors (comments about eating, weight of the partner). 

The results of this review confirm that social modeling of eating is a robust phenomenon. It occurs when the norm is set by another present person (i.e. another diner), but also when the model is not present, such as when the norm is communicated by textual information (list of amounts eaten by previous participants) [47]. Herman et al. [2] introduced Theory of Normal Eating which includes the three main factors that control food intake and choice—hunger, palatability, and social norms, but gives more weight to some (palatability and social norms) than to others (hunger). Social influence of eating is not restricted to "artificial" laboratory situations [48]. The number of chocolates taken by visitors to a work lunchroom was higher when the norm (empty chocolate wrappers in a bowl) indicated that other people had eaten the chocolates, than when there was no visible evidence of consumption [49]. In a study of Thomas et al. [50], posters containing a social norm message—information about vegetable purchases of other diners were placed in workplace restaurant. The results of these studies suggest that social norm messages may influence eating behaviors in real world settings.

Diverse policy interventions exist to address healthy eating. Health promotion campaigns and nutrition education benefits appear modest and effects usually reduce over time. Multi-component interventions appear to be more effective than single interventions [51]. Results of the umbrella review of Matwiejczyk et al. showed that in children, successful interventions were multi-component, multi-level and targeted both environmental and individual-level determinants of healthy eating behaviors [52]. Social marketing campaigns are developed for more precisely defined target audiences, it is important to utilize a wide range of theoretical frameworks in order to create campaigns that are best suited to meet the needs of diverse audiences [53]. One aspect of social marketing is the social norm approach [54]. Social norms strategies are intended to leverage differences between a person’s actual actions and attitudes and the person’s perceptions of what others do and think and correct misperceptions in the perceived social norms by publicizing the actual social norm [55]. Social norm marketing has been employed in environmentally-related domains (e.g. energy use, food waste) as well as health-related domains such as unhealthy lifestyle (e.g. alcohol use and cigarette smoking in young people) [56,57]. Interventions aimed at food choice and intake are scarce [58].

However, in the light of the results of the studies described above, using social norms approach in the implementation of interventions addressing unhealthy food choice and intake would seem to be a promising endeavor. Such intervention may be aimed at children and their parents because parent modeling is key to achieving healthy food choices [59]. Parents should be equipped with strategies enabling them to model healthy behaviors for their children and make the family meal environment conducive to nutritional health [60]. The input from the target group, the involvement of various stakeholders and close collaboration between researchers and those who will eventually implement social marketing campaigns is necessary for successful research and program delivery and translation and adoption of findings into practice [61,62].

A few limitations of research on social modeling of food intake should be mentioned. Firstly, a common feature of all early studies was the use of a taste-test paradigm. A methodological weakness is that these settings could be argued to be somewhat artificial. It is possible that because the participants were in a strange setting and unsure of how to complete the taste test, they may have been encouraged to follow the eating behavior of the confederate [63]. Results of a meta-analysis of Robinson et al. [64] of the effect of awareness of observation in laboratory settings suggest that it may reduce intake of participants. Researchers suggest that experiments on eating behavior may benefit from using methods that attempt to minimize participant awareness of observation, such as blinding participants to study aims, using cover stories, and/or making food consumption appear to be an incidental part of a study. Additionally, complementing laboratory studies with a more naturalistic real-world setting is recommended [64]. Secondly, the majority of social modeling studies have been conducted with predominantly normal-weight highly educated female participants, mainly psychology students. The homogeneity of the samples often limited the generalizability of the findings to other populations [65]. To assess this issue, research is conducted to replicate the results in different groups (e.g., men [17], restrained eaters [32], children [66], and different body weights [19]) Thirdly, most of the studies offered only one type of food, usually energy-dense snacks (e.g., cookies, M&M’s, or pizza) or limited kinds of vegetables. Some researchers suggested that their findings should be replicated with other types of snack food or complete meals [17,65].

We should also address some limitations of this narrative review. We did not provide a systematic search of articles published before 2015, as they were included in reviews by Vartanian et al. [5] and Cruwys et al. [6]. We decided to present descriptions of a variety of experiments in the field of social modeling of food intake and an update of studies published since abovementioned reviews. It brings the risk that we could have overestimated the value of some studies [67] and summarized the studies subjectively and narratively [68]. However, while systematic reviews are superior to narrative reviews in answering specific questions, narrative reviews are better suited to addressing a topic in wider ways [69]. 

Findings from this review and other studies [5,6] point to the fact that the social environment plays an important role in eating behaviors. The research in the field has practical implications: social modeling and social norms manipulations may be used to change people’s dietary practices, especially in children and young adults. Within the home environment parental modeling has been shown to promote children’s snacking [70] and fruit and vegetable consumption [71]. Parent’s dietary behavior has the impact upon children’s future diet [72]. Healthy eating interventions should thus include the family context and reinforce parents’ awareness of their influence on their children’s eating [73]. Parents should be encouraged to utilize the opportunity to model healthful dietary intake of fruit and vegetables at snacks [74]. Social modeling can be also used in food educational programs targeted at children. Young children may conform to food-related behaviors of remote peers; thus, social modeling may be used in interventions promoting healthier dietary choices of young children [75]. Children who viewed a video of peers consuming a vegetable ate more vegetable and reported greater preference for eating the vegetable again than children without video exposure [76]. Combined peer-modeling (video confederate—watching video depicting heroic peers who enjoyed eating fruit and vegetables) and reward-based intervention was associated with significant increase in children’s fruit and vegetable intake at school and at home [77]. Screen-based social modeling interventions may emerge as a low-cost, minimally invasive, and effective behavior change strategies to improve children’s vegetable consumption or as a tool in obesity prevention programs [76]. 

## Figures and Tables

**Table 1:** Characteristics of described experiments in order of appearance.

Ref	Year		Method	Ps	Sex	Variable	SM	Additional Outcome
[9]	1974		LC		M		Yes	
[10]	2001		RC	152	W	RC	Yes	
[11]	2013	Exp. 1 Exp. 2 Exp. 3	RC RC LC	71 112 93	W W W	Perceived norms		Perceived norms mediated modeling
[12]	2013		RC	64	W	RC	Yes	
[13]	2011		RC/LC	32	W	RC vs. LC		No difference between procedures
[14]	2009		VC	44	G	Participant’s body weight, VC	Yes	Modeling regardless of participant’s body weight
[15]	2012	Exp. 1 Exp. 2	VC	77 51	W W	VC	No	No modeling if VC was not in the same context
[16]	2018		VC	107	W	VC	Yes	
[17]	2010		LC	59	M	Hunger in men	Yes/No	Modeling only in hungry men
[18]	2002	Exp. 1	LC	48	W	Confederate’s body weight	Yes/No	No modeling if obese LC
[19]	2009	St. 2	LC	115	W	Confederate’s body weight	Yes	No modeling if obese LC
[20]	2015		RC	80	W	Remote confederate’s body weight	Yes	RC body weight did not moderate
[21]	2008		LC	102	W	Confederate’s body weight	Yes/No	Modeling only in normal weight LC
[22]	2009		LC	116	W	Healthy snacks	Yes	
[23]	2015	St. 1 St. 2	LC LC	129 120	W W	LC’s body weight, healthy snacks	Yes	Modeling of healthy snacks regardless of LC’s body weight
[24]	2019	St. 1 St. 2	RC RC	90 84	W W	Identification with a norm referent group, healthy snacks	Yes	Identification with the norm referent group did not moderate
[25]	2004	Exp. 1	RC	72	W	Palatable/unpalatable food	Yes/No	No modeling of unpalatable food
[26]	2021		D	51	W&M	Unfamiliar food	Yes	Partner’s presence increased consumption of unfamiliar food
[27]	1991	Exp. 1 Exp. 2	LC LC	86 63	W W	Hunger	Yes	Modeling is stronger than hunger
[28]	2015	Exp. 1 Exp. 2	RC RC	83 100	W W	Self-construal level	Yes	Participant’s self-construal level did not moderate
[29]	2015		D	178	W	Trait self-esteem	Yes	Trait self-esteem did not moderate
[30]	2019	St. 2	RC	122	W	Self-affirmation	Yes	Self-affirmation did not moderate
[31]	2020		VC	113	W&M	Psychological proximity/distance	Yes/No	Modeling only if primed with proximity
[32]	2020		RC+LC	735	W	Restrained/unrestrained eating	Yes	Restrained eaters were more responsive to the high-intake norm
[33]	2010	St. 1 St. 2	LC LC	54 47	W W	Weight-related cues	Yes/No	Modeling only when no weight-related cues
[34]	2013		LC	112	B&G	Role of emotions in children	Yes/No	Modeling only in happy and sad movie conditions, not in neutral
[35]	2015	St. 1 St. 2	D D	110 82	W	Familiarity of eating partners	Yes	Modeling regardless of familiarity of eating partners
[36]	2017		RC	108	W	Persistence and resistance to eating norms	Yes	Participant’s social influence history moderated
[37]	2018		LC	64	W	Non-eating LC	Yes	For some women, the effect of the non-eating confederate seemed to persist
[38]	2012		D	70	W	Mimicry	Yes	Mimicry was observed
[39]	2013		LC	68	B&G	Mimicry	Yes	Mimicry was observed
[40]	2015		D	38	G+W	Mimicry in adolescent-parent dyads	Yes	Mimicry was observed
[41]	2008	St. 1 St. 2	D D	122 75	W W	Awareness of social influence	Yes	People do not acknowledge social influence on their eating
[42]	2015	St. 1 St. 2	RC RC	80 80	W W	Awareness of social influence	Yes	People do acknowledge social influence on their eating

Abbreviations: Ref—reference; Ps—number of participants; SM—whether social modeling was observed; LC—live confederate; RC—remote confederate; VC—video confederate; D—dyad; W—women; M—men; G—girls; B—boys; St.—study; Exp.—experiment.

## Data Availability

Not applicable.
